# Genomic instability of human embryonic stem cell lines using different passaging culture methods

**DOI:** 10.1186/s13039-015-0133-8

**Published:** 2015-04-23

**Authors:** Lucie Tosca, Olivier Feraud, Aurélie Magniez, Cécile Bas, Frank Griscelli, Annelise Bennaceur-Griscelli, Gérard Tachdjian

**Affiliations:** AP-HP, Histologie-Embryologie-Cytogénétique, Hôpitaux Universitaires Paris Sud, Clamart, F-92141 France; Université Paris Sud, Le Kremlin-Bicêtre, F-94275 France; Esteam Paris Sud INSERM UMR-S 935, Villejuif, F-94801 France; Université Paris Descartes, Sorbonne Paris Cité, F-75006 France; AP-HP, Hématologie, Hôpitaux Universitaires Paris Sud, Villejuif, F-94801 France

**Keywords:** Cell passaging methods, Collagenase IV, Human embryonic stem cells, Array-CGH, Genomic instability

## Abstract

**Background:**

Human embryonic stem cells exhibit genomic instability that can be related to culture duration or to the passaging methods used for cell dissociation. In order to study the impact of cell dissociation techniques on human embryonic stem cells genomic instability, we cultured H1 and H9 human embryonic stem cells lines using mechanical/manual or enzymatic/collagenase-IV dissociation methods. Genomic instability was evaluated at early (<p60) and late (>p60) passages by using oligonucleotide based array-comparative genomic hybridization 105 K with a mean resolution of 50 Kb.

**Results:**

DNA variations were mainly located on subtelomeric and pericentromeric regions with sizes <100 Kb. In this study, 9 recurrent genomic variations were acquired during culture including the well known duplication 20q11.21. When comparing cell dissociation methods, we found no significant differences between DNA variations number and size, DNA gain or DNA loss frequencies, homozygous loss frequencies and no significant difference on the content of genes involved in development, cell cycle tumorigenesis and syndrome disease. In addition, we have never found any malignant tissue in 4 different teratoma representative of the two independent stem cell lines.

**Conclusions:**

These results show that the occurrence of genomic instability in human embryonic stem cells is similar using mechanical or collagenase IV-based enzymatic cell culture dissociation methods. All the observed genomic variations have no impact on the development of malignancy.

**Electronic supplementary material:**

The online version of this article (doi:10.1186/s13039-015-0133-8) contains supplementary material, which is available to authorized users.

## Background

Human embryonic stem cells (hESC) are derived from inner cell mass of blastocyst stage embryos [1]. These cells exhibit pluripotency and self-renewal properties. Indeed, hESC can differentiate into the three germlines that constitute a potential use of the cells in therapeutics, transplantation, drug testing as well as to study early embryogenesis. Even though it is possible to cultivate for a long time hESC and maintain their undifferentiated state the current *in vitro* culture conditions are not optimal and still new methods need to be developed. Previous studies showed that hESC could acquire nonrandom genetic changes after prolonged cell passages affecting cell growth and differentiation potential [2,3]. These genomic variations represent a selection providing to the cells a strong advantage [4,5]. The accumulation of chromosomal abnormalities during hESC culture may be due to decrease efficiency of base excision repair, surnumerary centrosomes and/or malfunction of the cell cycle checkpoints [5-8]. Environmental factors may influence genomic behavior such as culture media, feeder layer or dissociation methods used for cell passaging. Impact of cell culture dissociation techniques on genomic instability in particular enzymatic based-methods was underlined by many authors [2,9-15]. Others studies observed chromosomal integrity when mechanical/manual dissociation was used [16-18]. However, these studies were based on conventional cytogenetic techniques allowing a chromosomal study with a resolution of 10 Mb. Some authors combined these assays with chromosome based-comparative genomic hybridization (classic CGH) allowing an overview of the whole genome for the detection of DNA copy changes. But, similarly to the karyotype, resolution of classic CGH may not allow detection of genomic variations smaller than 10 Mb.

Recently, studies on hESC genomic instability were obtained using microarray-based comparative genomic hybridization (array-CGH) or single nucleotide polymorphisms (SNP) array allowing a high resolution chromosomal study under 100 Kb [19-22]. Nevertheless, the effects of passaging culture methods on genomic instability were not investigated using array-CGH.

In this study, we carried out an extensive molecular cytogenetic analysis on two hESC lines H1 and H9 cultured beyond 60 passages on mouse embryonic fibroblasts (MEF) and passaged by mechanical or collagenase IV-based enzymatic methods. We used array-CGH with a mean resolution of 50 Kb to uncover subkaryotypic genome alterations and subsequent gene content. Teratoma formation potency was realized in order to assess the functional impact of the dissociation methods used.

## Results

### Analysis of pluripotent markers expression across the time

During the successive cell passages, the hESC lines showed no morphological evolution that may indicate that they had lost their pluripotent status. However, to ensure their pluripotent state, we performed an expression analysis of three specific markers of pluripotence (HESCA, SSEA-4 and TRA 1–60) at early (<p60) and late (>p60) passages (Additional file 1: Figure S1). In all cases, we observed an extreme stability of expression of these three markers between early and late passages (Additional file 1: Figure S1). These results confirmed the maintenance of the pluripotent state of these cells during this study.

### Analysis of H1 and H9 hESC lines by conventional cytogenetic and FISH analysis

A systematic chromosomal assay using karyotype and FISH was realized at regular passages for the two cell lines H1 and H9 using both passaging methods (Additional file 2: Table S5). Analysis for H1 cell lines showed a normal male karyotype 46,XY (Additional file 3: Figure S2A). Similarly, H9 cell lines showed normal female karyotype 46,XX (Additional file 3: Figure S2B).

The centromeric probes specific for chromosomes 12 and 17 showed normal hybridization excluding chromosomes 12 and 17 aneuploidies. BACs RP5-1018D12 and RP3-324O17 hybridization gave one signal on both chromosomes 20 thus excluding the recurrent 20q11.2 duplication.

### Global characterization of genomic instability by array-CGH in H1 and H9 hESC lines

**Figure 1:**
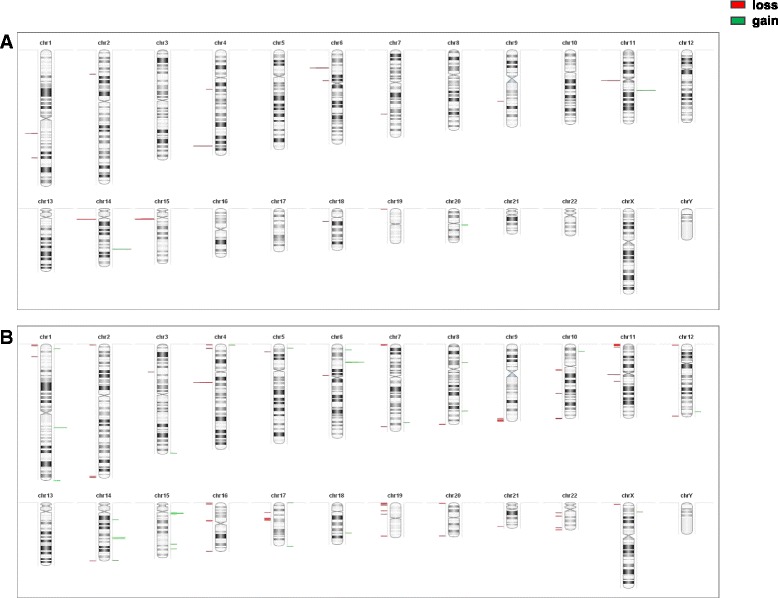
Array-CGH DNA variations distribution. Individual distribution of total chromosomal aberrations for H1 **(A)** and H9 **(B)** hESC cell lines including polymorphic CNVs described in Toronto Database of Genomic Variants. Each bar represented one genomic variation: green, gain; red, loss.

First we analyzed array-CGH results considering all genomic variations i.e. including polymorphic DNA variations described in Toronto Database of Genomic Variants (http://projects.tcag.ca/cgi-bin/variation/gbrowse/hg18/). Individual representation of genomic variations of the two hESC lines is shown in Figure 1. We observed that the genomic variations were mainly located on subtelomeric and pericentromeric regions.

Table 1 mentions 9 recurrent DNA variations occurring during culture of the hESC lines. These genomic variations are described according to chromosomal position, size, gene content and type (gain/loss). We observed that more than half-variations (6/9) carried relevant genes involved in development, cell cycle, growth, apoptosis, tumorigenesis and/or syndrome/disease (Table 1). An example of plots of the 1q21.3 region is presented in Additional file 4: Figure S3.

**Table 1:** **Recurrent genomic variations acquired during hESC H1 and H9 culture**

**Chromosome region**	**Size (kb min-max)**	**Common genes**	**H1 M early**	**H1 E early**	**H1 E late**	**H9 M early**	**H9 E early**	**H9 E late**
1q21.3	12.42-25.84	LCE3C^4^	L	-	L	G	-	G
4p16.3	27.74-253.75	FAM53A	-	-	-	-	G	L
6p21.32	37.12-81.43	HLA-DRB5^4^	L	L	L	G	G	G
11q11	54.62-82.97	OR4C11, OR4P4, OR4S2	L	L	L	L	-	L
14q23.2	295.35	KCNH5, RHOJ^3^, GPHB5	-	-	-	G	-	G
14q23.3	398.69	FUT8^3^	-	-	-	G	-	G
15q11.2	1270.33-1779-68	LOC283755, A26B1, OR4M2, OR4N4, LOC650137	L	L	L	G	-	G
20q11.21	1022.42	DEFB115-116,118-119,121,123-124, REM1, HM13, ID1^2^, COX4I2^4^, BCL2L1^2^	-	-	G	-	-	-
22q13.2	17.66	SCUBE1^1^	-	-	-	-	-	L

Recurrent genomic variations observed during hESC lines H1 and H9 *in vitro* culture using manual or enzymatic techniques. Chromosomal region, minimal/maximal sizes, common genes and variation type (loss/gain) are mentioned. E, enzymatic passages; G, gain; L, loss; M, manual passages; max, maximal; min, minimal; ^1^, genes related to development; ^2^, genes related to cell cycle, growth and apoptosis; ^3^, genes related to tumorigenesis; ^4^, genes related to syndrome and disease.

**Figure 2:**
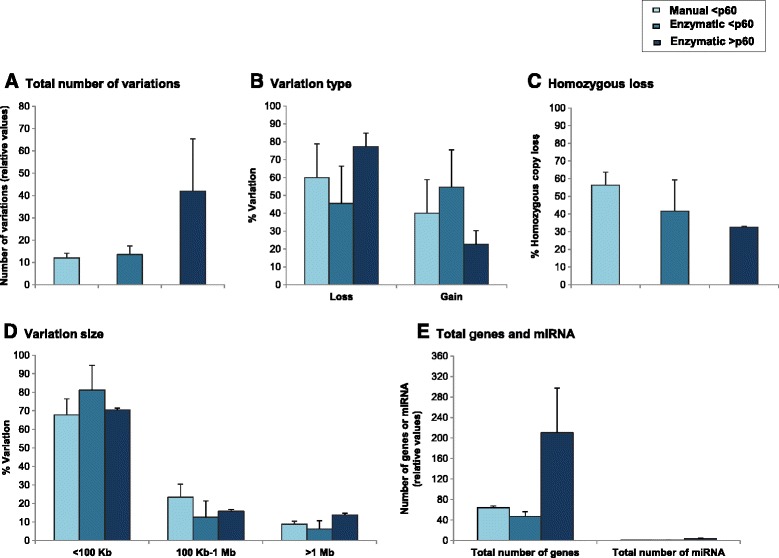
array-CGH DNA variations including polymorphisms at early manual, early enzymatic and late enzymatic passages. Comparison of genomic variations including polymorphic CNVs. Results are presented as mean of the two cell lines (early manual passages, H1p56, H9p30; early enzymatic passages, H1p56, H9p30; late enzymatic passages, H1p159, H9p87). The criteria evaluated were total number of variations **(A)**, variation type (gain/loss) **(B)**, homozygous loss percentage **(C)**, variation size (<100 Kb, 100 Kb-1 Mb, >1 Mb) **(D)**, and total number of genes and miRNA **(E)**.

Detailed characteristics of total genomic variations comparing early (**<**p60) manual, early enzymatic (**<**p60) and late enzymatic (**>**p60) passages are shown in Figure 2. Results are presented as mean of the two cell lines (early manual passages, H1p56, H9p30; early enzymatic passages, H1p56, H9p30; late enzymatic passages, H1p159, H9p87). Total number of genomic variations was stable after enzymatic technique (13.50 ± 3.88) compared to manual technique (12.00 ± 2.12) at early passages; and increased by about 3-fold after late enzymatic passages (42.00 ± 23.33) compared to early enzymatic passages (13.50 ± 3.88) but were not significantly different (Figure 2A). There were no significant differences between the percentages of DNA losses or DNA gains between early manual and early enzymatic passages (loss, 60.00 ± 18.85% *versus* 45.39 ± 20.93%; gain, 40.00 ± 18.85% *versus* 54.60 ± 20.93%) and between early and late enzymatic passages (loss, 45.39 ± 20.93% *versus* 77.33 ± 7.54%; gain, 54.60 ± 20.93% *versus* 22.66 ± 7.54%) (Figure 2B). The percentages of homozygous copy loss were not significantly different between both cell dissociation methods at early passages (early manual, 56.41 ± 7.25%; early enzymatic, 41.66 ± 17.67%) and between enzymatic passages (early enzymatic, 41.66 ± 17.67%; late enzymatic, 32.57 ± 0.53%) (Figure 2C).

When looking at the size of the variations, we observed no significant differences between early manual and early enzymatic passages or early enzymatic and late enzymatic passages (Figure 2D). However, for each group small genomic variations <100 Kb were more importantly observed than intermediate genomic variations (100 Kb-1 Mb) and than large genomic variations (>1 Mb) (Figure 2D). Indeed, for early manual group small genomic variations were about 3-fold higher than intermediate genomic variations (67.77 ± 8.64% *versus* 23.33 ± 7.07%) and by about 7.5-fold higher than large genomic variations (67.77 ± 8.64% *versus* 8.88 ± 5.97%) (Figure 2D). For early enzymatic group, small variations were about 6.5-fold higher than intermediate variations (81.25 ± 13.25% *versus* 12.50 ± 8.83%) and by about 13-fold higher than large variations (81.25 ± 13.25% *versus* 6.25 ± 4.41%) (Figure 2D). For late enzymatic group, small variations were about 4.5-fold higher than intermediate variations (70.44 ± 10.52% *versus* 15.77 ± 1.76%) and by about 5-fold higher than large variations (70.44 ± 10.52% *versus* 13.77 ± 5.97%) (Figure 2D).

Total number of genes included in genomic variations was not significantly different between early manual and early enzymatic passages (64.00 ± 3.53 and 47.00 ± 9.19, respectively) and between early enzymatic and late enzymatic passages (47.00 ± 9.19 and 210.5 ± 86.62, respectively) (Figure 2E). The number of miRNA included in genomic variations was quite similar between early manual (1.00 ± 0.00) and early enzymatic (0.50 ± 0.35) methods; and between early (0.50 ± 0.35) and late (3.50 ± 1.06) enzymatic methods (Figure 2E).

### Characterization of genomic variations without polymorphic CNVs by array-CGH of H1 and H9 hESC cell lines

**Figure 3:**
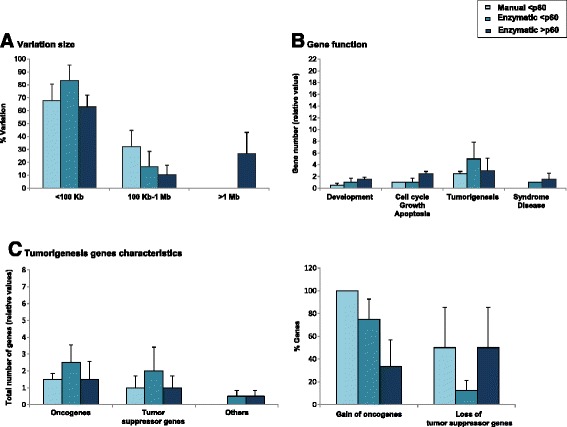
Array-CGH DNA variations without polymorphisms at early manual, early enzymatic and late enzymatic passages. Comparison of genomic variations excluding polymorphic CNVs. Results are presented as mean of the two cell lines (early manual passages, H1p56, H9p30; early enzymatic passages, H1p56, H9p30; late enzymatic passages, H1p159, H9p87). The criteria evaluated were variation size **(A)**, gene content distributed in 4 groups according to their function: development, cell cycle/growth/apoptosis, tumorigenesis and syndrome/disease **(B)**, and tumorigenesis gene characteristics **(C)**.

Secondly, we interpreted array-CGH results without polymorphic variants described in Toronto Database of Genomic Variants. The content of this database is only representing structural variation identified in healthy control samples. Thus remaining DNA variation can be interpreted as potentially pathogenic or of unknown signification. Comparison between early manual (**<**p60)*,* early enzymatic (**<**p60) and late enzymatic (**>**p60) passages is shown in Figure 3. Results are presented as mean of the two cell lines (early manual passages, H1p56, H9p30; early enzymatic passages, H1p56, H9p30; late enzymatic passages, H1p159, H9p87).

Concerning the size of DNA variations, we did not observe significant differences between early manual and early enzymatic methods for small genomic variations <100 Kb (67.85 ± 12.62% *versus* 83.33 ± 11.78%), intermediate genomic variations 100 Kb-1 Mb (32.14 ± 12.62% *versus* 16.66 ± 11.78%) and large genomic variations (0.00 ± 0.00% *versus* 0.00 ± 0.00%) (Figure 3A). Similarly, we did not observe significant differences between early enzymatic and late enzymatic methods for small (83.33 ± 11.78% *versus* 62.93 ± 9.14%), intermediate (16.66 ± 11.78% *versus* 10.34 ± 7.31%) and large (0.00 ± 0.00% *versus* 26.72 ± 16.46%) genomic variations (Figure 3A). However, for each group variations <100 Kb were more importantly observed. Indeed, for early manual group, small variations were about 2-fold higher than intermediate variations (67.85 ± 12.62% *versus* 32.14 ± 12.62%) and higher than large variations (67.85 ± 12.62% *versus* 0.00 ± 0.00%, P = 0.06) (Figure 3A). For early enzymatic group, small variations were about 5-fold higher than intermediate variations (83.33 ± 11.78% *versus* 16.66 ± 11.78%) and higher than large variations (83.33 ± 11.78% *versus* 0.00 ± 0.00%) (Figure 3A). For late enzymatic group, small variations were about 6-fold higher than intermediate variations (62.93 ± 9.14% *versus* 10.34 ± 7.31%) and by about 4-fold higher than large variations (62.93 ± 9.14% *versus* 26.72 ± 16.46%) (Figure 3A).

**Figure 4:**
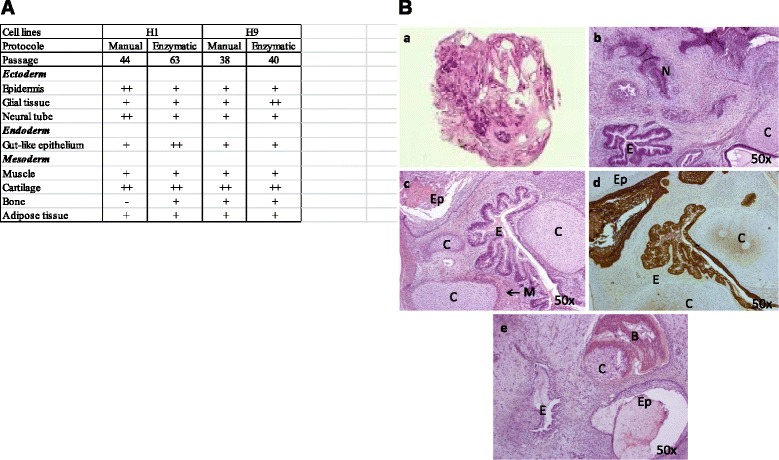
Germ cell layers components within teratomas. Differentiation of H1 and H9 (picked at different passages and dissociated by manual or enzymatic protocols) into ectoderm, endoderm and mesoderm was quantified using whole section Hematoxylin and eosin stains **(A)**. All lines showed structures representing the 3 lineages (ectoderm, endoderm and mesoderm) and were scored as follows: “ - ” indicating the absence of the tissue; “ + ” and “ ++ ” indicating the existence of differentiated tissues that were respectively moderately (one to 5 focus) or highly (>5 focus) represented. Characteristics of hESC-derived teratomas **(B)**: a, b, c; Hematoxylin and eosin staining of teratoma derived from H1 (passage 63 and treated with enzymatic dissociation). d; Cytokeratin AE1/AE3 immunostaining of teratoma derived from H1 showing epithelial structures. e; Hematoxylin and eosin staining of teratoma derived from H9 (passage 40 and treated with enzymatic dissociation). N, neural tissue (ectoderm); C, cartilage (mesoderm); Ep, keratin-containing epidermal tissue (ectoderm); M, striated muscle (mesoderm); B, bone (mesoderm); E, gut epithelial tissue (endoderm).

For genes carried by genomic variations, we did not observe significant increase of gene number in early enzymatic group *versus* early manual group independently of what the gene functions are (development, 1.00 ± 0.70 *versus* 0.50 ± 0.35; cell cycle/growth/apoptosis, 1.00 ± 0.70 *versus* 1.00 ± 0.00; tumorigenesis, 5.00 ± 2.82 *versus* 2.50 ± 0.35; syndrome disease 1.00 ± 0.00 *versus* 0.00 ± 0.00); nor in late enzymatic *versus* early enzymatic groups (development, 1.50 ± 0.35 *versus* 1.00 ± 0.70; cell cycle/growth/apoptosis, 2.50 ± 0.35 *versus* 1.00 ± 0.70; tumorigenesis, 3.00 ± 2.12 *versus* 5.00 ± 2.82; syndrome disease 1.50 ± 1.06 *versus* 1.003 ± 0.00) (Figure 4B). The list of genes involved in development; cell cycle, growth and apoptosis; tumorigenesis; and syndrome and disease are presented in Additional file 5: Table S1, Additional file 6: Table S2, Additional file 7: Table S3 and Additional file 8 Table S4, respectively.

When looking at tumorigenesis genes, we did not observe differences between early enzymatic *versus* early manual groups for total number of oncogenes, of tumor suppressor genes or of other genes like fusion genes (oncogenes, 2.50 ± 1.06 *versus* 1.50 ± 0.35; tumor suppressor genes, 1.00 ± 0.70 *versus* 2.00 ± 1.41; others, 0.50 ± 0.35 *versus* 0.00 ± 0.00); nor between late enzymatic *versus* early enzymatic groups (oncogenes, 1.50 ± 1.06 *versus* 2.50 ± 1.06; tumor suppressor genes, 1.00 ± 0.70 *versus* 2.00 ± 1.41; others, 0.50 ± 0.35 *versus* 0.50 ± 0.35) (Figure 4C, left panel). Similarly, the percentage of oncogenes gain and the percentage of tumor suppressor genes loss were not different between early enzymatic *versus* early manual groups (75.00 ± 17.67% *versus* 100.00 ± 0.00% and 12.50 ± 8.83 *versus* 50.00 ± 35.35, respectively) nor between late *versus* early enzymatic groups (33.33 ± 23.57% *versus* 75.00 ± 17.67% and 50.00 ± 35.35 *versus* 12.50 ± 8.83, respectively) (Figure 4C, right panel).

### Evaluation of the differentiation potential of H1 and H9 cell lines *in vivo*

Teratoma-forming potential and tumor content by histological analysis were explored in the H1 and H9 cell lines. This teratoma assay enabled clear-cut evaluation of the impact of both dissociation protocols on the behaviour, the differentiation and the proliferation of hESCs *in vivo* over a period of several months. Furthermore, the teratoma assay is the only test that may reveal the tumoral potential linked with hESC that have acquired genome alterations and specially genes involved in tumorigenesis or in specific syndrome and disease. For the teratoma assay, 2 to 3x10^6^ cells were picked at early (p38/p40/p44) and late (p63) passages were injected intramuscularly. For H1 and H9 the two dissociation protocols were compared (Figure 4A). All four hESC produced teratomas after 96 to 160 days. Histological analysis showed a differentiation into ectodermal, endodermal and mesodermal tissues, mainly represented by glial tissues, glandular epitheliums and large cartilaginous areas, respectively (Figure 4B). All the structures observed showed mature and well differentiated tissues without malignancy. Furthermore all teratomas were negative for Ki-1 antigen (CD30) (data not shown) which is highly predictive of pluripotent stem cells-derived malignancies [23], confirming the absence of any embryonic malignant tissues.

In conclusion, for the two hESC lines H1 and H9, global genomic variations including polymorphisms were particularly observed in subtelomeric and pericentromeric regions and were mainly of small size (<100 Kb). The use of collagenase IV did not induced significant differences. When excluding polymorphic genomic variations during data interpretation, we found no effect on gene content neither between early manual *versus* early enzymatic passages nor between early enzymatic *versus* late enzymatic passages. Furthermore, both protocols had no functional effect on *in vivo* malignancy as demonstrated by teratoma assays.

## Discussion

Our results showed that during short and long term culture of the hESC H1 and H9, DNA variations were mostly located on instable regions of the genome such as subtelomeric and pericentromeric regions. In our study, the use of collagenase IV for cell dissociation did not induce significant changes on genes carried by these genomic variations in comparison with mechanical cell passages.

Genomic variations include single nucleotide polymorphisms (SNP), insertions/duplications, deletions and inversions/translocation. Variability of copy number variation (CNV) is estimated to 10-20% of the human genome ([24,25], Database of Genomic Variants http://projects.tcag.ca/cgi-bin/variation/gbrowse/hg18/). The genomic variations are considerated as either polymorphic (or benign, currently referenced in Toronto database of genomic variants), of unknown signification or pathogenic. In our study, we found a mean of twenty-four CNV per hESC line analyzed. The size of CNV ranged from 0.4 Kb to 3.155 Mb, i.e. undetectable using standard karyotype analysis (mean resolution of 10 Mb). We found more small genomic changes (<100Kb) than intermediate (100 Kb-1 Mb) and large (>1 Mb) ones. Larger genomic changes have a greater chance of conferring a negative advantage, and of being selected out. We observed that DNA variations were carried by instable regions of the genome such as subtelomeric and pericentromeric regions. Subtelomeric and pericentromeric regions are dynamic chromosomal regions carrying repetitive elements. Other chromosomal regions are considered instable in relation to the architecture of the genome as segmental duplications. These particular conformations of DNA favor deletion and duplication aberrations. Pathogenic CNV may affect gene expression and may influence phenotypic variation by disrupting genes and altering dosage compensation. Thus, DNA copy variations may influence replication/proliferation, differentiation and functional potentials of human ESC.

In the past few years, several authors reported controversial results on genomic integrity during prolonged *in vitro* hESC culture [2,3,11-16,26-29]. These results may be dependent on the passaging methods used, on the use or not of feeders or on the duration of the culture. Indeed, according to *in vitro* culture systems, selective pressures from the environment may influence genomic stability. However, genomic stability of hESC lines is essential for maintaining cell properties [30]. Some studies on hESC genomic instability using array-CGH or SNP array were recently published however the effect of passaging method was not investigated [19-21].

It is important to distinguish random and nonrandom DNA variations. Nonrandom acquired somatic mutations reflect a selective advantage independent of culture conditions. Genomic changes affecting others regions might be more likely to depend upon culture conditions. The International Stem Cell Initiative consortium observed a large differential in frequency between gain and loss of chromosomes, gains being more frequent [20]. Aberrations are located in all chromosomes except chromosome 4 [20]. Some abnormalities such as gain of chromosome 12 (presence of the pluripotency *NANOG* gene and the cell-cycle regulator *CCND2* in 12p13; the oncogene *KRAS* in 12p12.1), chromosome 17 (*BIRC5* candidate gene in 17q25, an antiapoptotic gene associated with the highest-risk tumor), chromosome 20 and chromosome X or fragments of these chromosomes may promote self-renewal and thus provide a selective proliferative/survival advantage [3,31,32]. Nonrandom abnormalities revealed culture adaptation of human ES as suggested by the ISCI consortium study [20]. ISCI meta-analysis revealed a recurrent loss in 22q13-qter region, as a novel finding. In contrast, other karyotypic abnormalities result in no effect or delayed cell cycle and proliferation rate.

Recurrent genomic variations, as illustrated in Table 1, were observed in the two unrelated hESC lines. This observation suggests that some specific chromosomal regions confer a selective advantage to the cells during *in vitro* culture. In accordance with published results, we found recurrent gain of 1q21, 20q11.2 and loss of 22q13 regions (Table 1) [4,5,20,31-35]. These three chromosomal regions harbored genes of particular importance for culture adaptation like *LCE3C* (involved in barrier repair after injury or inflammation, 1q21.3; [36]), *BCL2L1* (regulates cell survival and death, 20q11.21; [37]) and *SCUBE1* (roles in development, inflammation and thrombosis, 22q13.2; [38]). We found that copy number variation (deletion or duplication) of the 6p21.32 region was systematically detected. The minimal region included the *HLA-DRB5* gene, coding for HLA class II beta chain paralogues. The *HLA-DRB5* gene is involved in genetic susceptibility to multiple sclerosis (MS) as *HLA-DRB5* null subjects appear to be at increased risk for developing secondary progressive MS [39]. Wu and colleagues also described a deletion 6p21.32 of 40 Kb including this gene in HSF1 and HSF6 hESC lines using 244 K array-CGH [33]. The 20q11.21 amplification was described by many authors [4,5,12,21,33,40].

By using array-CGH with a mean resolution of 50 Kb, we failed to detect any significant effect of the passaging method used on hESC genomic instability when comparing manual/mechanical *versus* enzymatic/collagenase IV methods. However, it cannot be excluded that genomic instability below 50 Kb could have occurred. This could have been detected by other sensitive methods such as deep genomic sequencing. To our knowledge, published data are still controversial. Caisander and colleague used in their retrospective study karyotype, fluorescent *in situ* hybridization (FISH) and classic CGH on five hESC lines (SA002, SA002.5, AS034.1.1, SA121 and SA461) after prolonged *in vitro* culture [16]. When using mechanical cell dissociation method, the authors concluded on chromosomal integrity even after two freeze-thaw procedures and 148 passages of cell culture. Catalina study in 2009 used conventional karyotype, spectral karyotyping (SKY), interphase FISH and classic CGH on two hESC lines (HS181, HS293) [17]. The authors concluded on an overall genomic stability whilst maintaining hESC properties (typical morphology, transcription factors and markers expression associated to undifferentiated status and *in vitro/in vivo* pluripotency). However, mechanical cell dissection combined or not with enzymatic methods may affect cell growth [10,41-44]. During manual passages, colonies are cut into small pieces related to their morphology in contrast to enzymatic methods. However, enzymatic passaging is more efficient in generating sufficient numbers of undifferentiated cells.

An important issue is to evaluate if the genomic alteration and the recurrent genomic variations, which appeared in hESC after repeated passages (with manual or enzymatic techniques) have a strong impact on their differentiation and in tumorigenesis potentials. For this purpose we generated 4 teratomas with hESC picked at early passages. We were able to show that the tested hESCs H1 and H9 had the capacity to develop a complete teratoma displaying tissue from all three germ layers. In addition, we did not find any malignant tissue within the teratoma demonstrating that all the observed genomic variations have no impact on the development of malignancy. We also performed a Ki-1 antigen (CD30) immunostaining that was shown previously to be highly predictive of pluripotent stem cells-derived malignancies [23]. All teratomas were negative for CD30 confirming the absence of any embryonic malignant tissues.

## Conclusions

In conclusion, based on our observations we can advocate the utilization of collagenase IV for hESC *in vitro* culture cell dissociation, a less time-consuming method, compared to the manual passaging method. Indeed, there is no difference on cell genomic integrity at early passages (<p60). Furthermore, regardless of the dissociation protocol used, teratoma formation was not associated with embryonic malignant tissues. Our study also showed 9 recurrent copy number variations occurring in hESC culture including the well-known duplication 20q11.21.

## Methods

### Human Embryonic Stem Cell (hESC) lines and culture

All the experiments were approved by the French Biomedical Agency and conducted under the agreement # RE07-008R. Human embryonic stem cell lines H1 and H9 derived from 46,XY and 46,XX embryos, respectively (Wicell Research Institute, http://www.wicell.org) were maintained into undifferentiated state by continuous culture on a feeder layer of mitomycin C inactivated mouse embryonic fibroblasts in DMEM/F12 supplemented with 20% Knock Out Serum Replacer, 1 mM L-glutamine, 0.5% penicillin/streptomycin, 100 μM 2-mercaptoethanol and 10 ng/ml basic FGF (all of them from Invitrogen, Saint-Aubin, France). Cells were weekly passaged by a mechanical method using a 190–210 μm glass pipette (Stem cell cutting tool, Swemed, Bayonne, France) under a stereomicroscope (Lynx, Fisher Bioblock, Strasbourg, France) or by an enzymatic treatment followed by mechanical dissociation (“enzymatic method”). Manual techniques were not performed beyond 60/65 passages. The enzymatic passaging was carried out by incubating the cells in 1 mg/ml collagenase IV in DMEM/F12 (Invitrogen, Saint-Aubin, France) during 90 min at 37°C and 5% CO_2_. Colonies were then collected and pelleted by gravity. After two cycles of washing/sedimentation with DMEM/F12 alone, colonies were mechanically disrupted by 10 times pipetting with a 1 ml micropipette. Additional file 2: Table S5 summarized the passages at which further analysis were realized.

### Flow cytometry

Undifferentiated hESC colonies were recovered from the MEF by incubation in 1 mg/ml collagenase IV in DMEM/F12 followed by two washes in phosphate buffer saline (Invitrogen, Saint-Aubin, France). Before staining, recovered colonies were incubated in Hank’s based enzyme free cell dissociation buffer (Invitrogen, Saint-Aubin, France) 10 min at 37°C and dissociated to the single cell level by 10 times pipetting with a 1 ml micropipette. Cells were stained using FlowCellect Human ESC (HESCA-1, SSEA-4) Surface Marker Characterization kit (Millipore, Molsheim, France) or PE-conjugated mouse anti-human TRA 1–60 (clone TRA 1–60, BD Biosciences, Le Pont de Claix, France) according to manufacturer’s instructions and analyzed on a MACSQuant flow cytometer (Miltenyi Biotec, Paris, France) with MACSQuantify software (Miltenyi Biotec, Paris, France).

### Conventional cytogenetic analysis

Chromosome analyses by standard karyotype were performed from 30 cultured cells using standard procedures (R-bands by heating using Giemsa or RHG, and G-bands by trypsin using Giemsa or GTG bandings) at regular passages. The passage number listed represents the total passage number of the cell line at the time of analysis. For mitotic preparations, cells were cultured in DMEM/F12 supplemented with 0.02 mg/ml colchicin (Eurobio, Courtaboeuf, France) for up to 1 h and 45 min. The cells were harvested and warm hypotonic solution of 0.075 M KCl was added in the preparation for up to 15–20 min. Finally, the cells were fixed several times in cold Carnoy’s fixative (methanol/acetic acid, 3:1).

### Fluorescent *in situ* hybridization (FISH)

FISH analyses were performed on 100 interphasic nuclei and 20 metaphase spreads from H1 and H9 cell lines at regular passages. The centromeric probes specific for chromosome 12 and 17 were used according to manufacturer’s recommendations (Vysis, Downers Grove, IL). BAC clones specific for the 20q11.2 chromosomal region (RP5-1018D12 and RP3-324O17) were used (Bluegnome, Cambridge, UK).

### DNA extraction

Genomic DNA from the two hESC lines was isolated using a DNeasy Blood and Tissu Kit (Qiagen, Courtaboeuf, France). The extracted DNA concentrations were estimated using a NanoDrop ND-1 000 spectrophotometer (NanoDrop Technologies, Wilmington, DE, USA). Extracted DNA was used for array-CGH.

### Oligonucleotide array-comparative genomic hybridization (array-CGH)

The genomic imbalances of the two hESC lines were analyzed by array-CGH using 105 K oligonucleotide arrays (Hu-105A Agilent Technologies, Massy, France) at early manual passages (<60 passages; H1p56, H9p30), at early enzymatic passages (<60 passages; H1p56, H9p30) and at late enzymatic passages (>60 passages; H1p159, H9p87). The passage number listed represents the total passage number of the cell line at the time of analysis. All array hybridizations were performed according to the manufacturer’s recommended protocols. Briefly, 2 μg of genomic DNA were digested with AluI (5 units) and RsaI (5 units) for 2 h at 37°C and fluorescently labeled with the Agilent Genomic DNA labeling kit PLUS (Agilent Technologies, Massy, France). Male or female human genomic DNA (Promega, Charbonnière, France) was used as reference. Experiments were done in dye-swap. Cy5-dUTP cell line DNA and gender-matched reference DNA labeled with Cy3-dUTP were denatured and preannealed with Cot-1 DNA and Agilent blocking reagent prior to hybridization for 40 h at 20 rpm in a 65°C rotating hybridization oven (Agilent Technologies, Massy, France). After washing, the slides were scanned on an Agilent Microarray Scanner. Captured images were processed with Feature Extraction 9.1 software and data analysis was performed with DEVA software v1.0.2 (Roche Nimblegen, Meylan, France). The Nexus Copy Number Standard edition software (Proteigene, Saint-Marcel, France) algorithm was used for statistical analysis according to the version 18 of the Human genome built (http://genome.ucsc.edu/). CNV were considered significant if they were defined by 3 or more oligonucleotides spanned at least 50 Kb. The detection limit for mosaicism rate was arround 10% as recommended and previously described [45].

### Teratoma formation and immunohistochemistry

The teratoma assay was performed with hESC lines (H1 and H9) by intra-muscular injection of 2 to 3×10^6^ of cells into 6-week-old NOD/SCID mice (Charles River Laboratories). H1 cells were injected after 44 and 63 passages performed by mechanical or enzymatic dissociation respectively. H9 cells were injected after 38 and 40 passages performed by mechanical or enzymatic dissociation respectively. After 96 to 160 days, the teratomas were dissected and fixed in 4% paraformaldehyde and samples were embedded in paraffin and stained with hematoxylin and eosin in order to assess the presence of ectodermic, endodermic and mesodermic tissues or the presence of malignant tissue. Immunohistochemistry was performed as requested with a Benchmark XT apparatus (Ventana, France) with prediluted primary antibodies raised against AE1/AE3 (anti-pan-cytokeratin, BD Biosciences, France), and Ki-1 antigen CD30 (Ventana).

To evaluate the intensity of CD30-positive areas, an image of the whole teratoma was taken with a digital camera (PCO, Germany) and analyzed by the pathologist. For each histological image, CD30-positive areas and the total surface of the teratoma were manually selected under Adobe Photoshop. The CD30 expression was analyzed in extracts of the different teratomas by using Western blot analysis, as previously described [46]. Briefly, 40 μg of protein was loaded in each well, and the nitrocellulose membrane was probed with goat anti-CD30 antibody (1:200 Santa cruz-1737). Goat anti-rabbit peroxidase-linked antibody (1:10,000; Promega) was used as a secondary antibody. Actin was detected with monoclonal anti-actin peroxidase conjugated antibody (1:25,000; Sigma-Aldrich).

### Statistical analysis

All array-CGH experimental data are presented as mean ± SEM. The t-test relative frequencies for independent variables were used to test the differences. Statistical analysis was performed using BiostaTGV online software (http://marne.u707.jussieu.fr/biostatgv/). We compared i. early manual passages *versus* early enzymatic passages ii. early enzymatic passages *versus* late enzymatic passages. The differences were assumed to be significant when P-value <0.05.

## Additional files

Additional file 1: Figure S1. Pluripotency markers expression. Flow cytometric analysis of HESCA1, SSEA-4 and TRA 1-60 pluripotency markers expression by H1 at passages 61 (A) and 139 (B), H9 at passages 35 (C) and 69 (D). Additional file 2: Table S5. Passage number at which analysis were done in hESC lines H1 and H9. List of the passage number using either manual or enzymatic (collagenase IV) methods at with flow cytometry, karyotype, array-CGH and teratoma analysis were realized of the hESC lines H1 and H9. Additional file 3: Figure S2. Conventional cytogenetic analysis. A. G-banded karyotype of H1 hESC line at passage 64 showing a normal male karyotype 46,XY. B. G-banded karyotype of H9 hESC line at passage 59 showing a normal female karyotype 46,XX. Additional file 4: Figure S3. Chromosome 1 profile from array-CGH analysis showing recurrent variation on 1q21.3 region. Profiles are illustrated for H1p56 (a; early and manual passage), H1p159 (b; late and enzymatic passage), H9p30 (c; early and manual passage) and H9p87 (d; late and enzymatic passage). For each sample, whole chromosome 1 profile showing the interstitial variation is presented on the left panel; and the 1q21.3 deletion (a and b) or duplication (c and d) is zoomed in the right panel. Additional file 5: Table S1. Genes related to development. List of genes related to development located in genomic variations corresponding to Figure 3B. Chromosomal position, gene symbol and encoded protein are noted. G, gain; L, loss. Additional file 6: Table S2. Genes related to cell cycle, growth and apoptosis. List of genes related to cell cycle, growth or apoptosis located in genomic variations corresponding to Figure 3B. Chromosomal position, gene symbol and encoded protein are noted. G, gain; L, loss. Additional file 7: Table S3. Genes related to tumorigenesis. List of genes related to tumorigenesis located in genomic variations corresponding to Figures 3B and 3C. Chromosomal position, gene symbol and encoded protein are noted. G, gain; L, loss. Additional file 8: Table S4. Genes related to syndrome and disease. List of genes related to syndrome or human disease located in genomic variations corresponding to Figure 3B. Chromosomal position, gene symbol and encoded protein are noted. G, gain; L, loss.

## Abbreviations

array-CGH: microarray-based comparative genomic hybridization
CNV: Copy number variation
FISH: Fluorescent *in situ* hybridization
GTG: G-bands by trypsin using Giemsa
hESC: Human embryonic stem cells
MEF: Mouse embryonic fibroblasts
RHG: R-bands by heating using Giemsa
SKY: Spectral karyotyping
SNP: Single nucleotide polymorphisms
