# Supplementary figures and images for: Genomic instability of human embryonic stem cell lines using different passaging culture methods
Source: Mol Cytogenet. 2015 Apr 23;8:30. doi: 10.1186/s13039-015-0133-8 (PMC4456787; doi:10.1186/s13039-015-0133-8)

## Slide 1
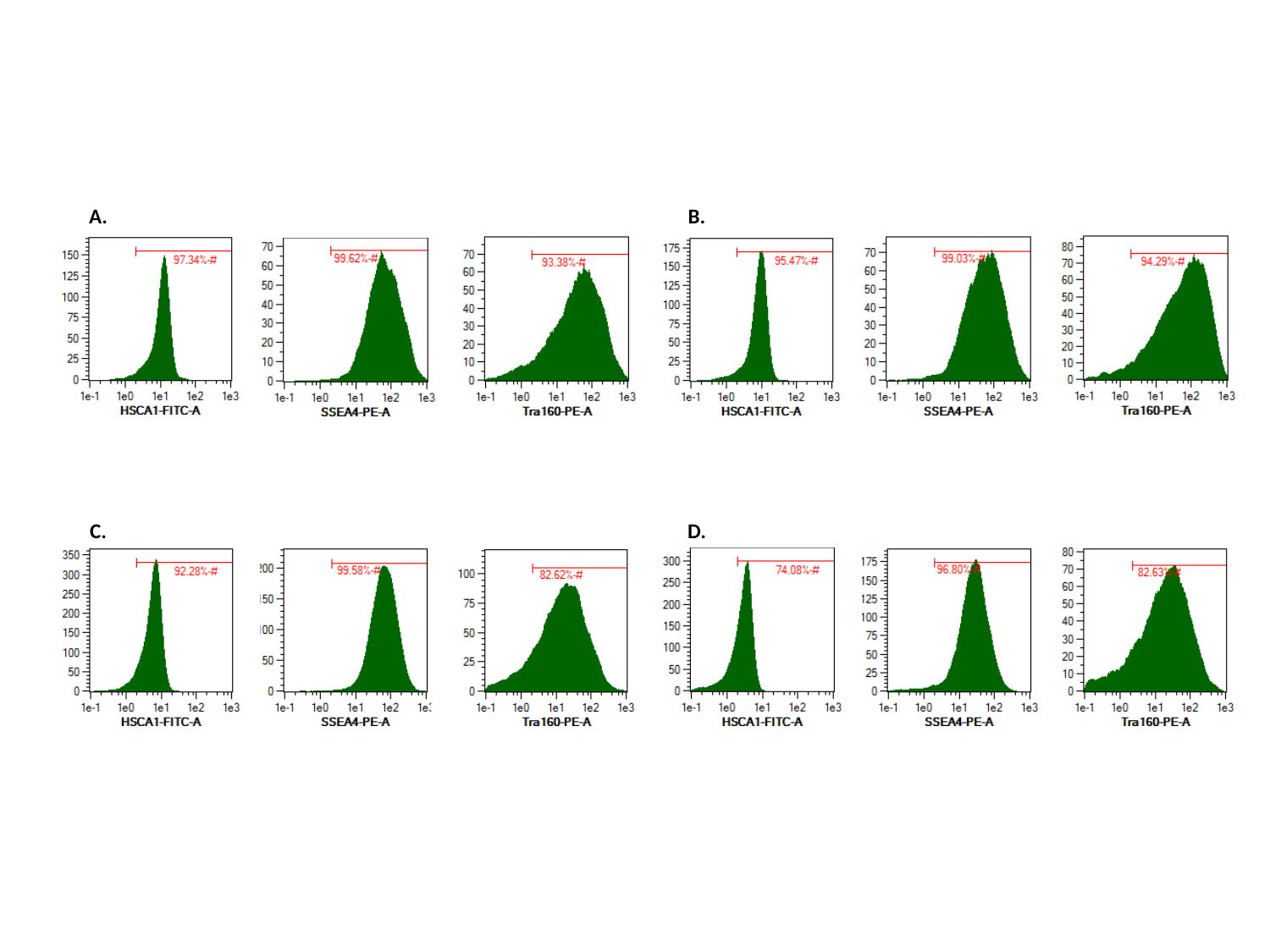

A.
B.
C.
D.

Supplement: Additional file 1: Figure S1. — Pluripotency markers expression. Flow cytometric analysis of HESCA1, SSEA-4 and TRA 1-60 pluripotency markers expression by H1 at passages 61 (A) and 139 (B), H9 at passages 35 (C) and 69 (D). [file 13039_2015_133_MOESM1_ESM.pptx]

## Slide 1
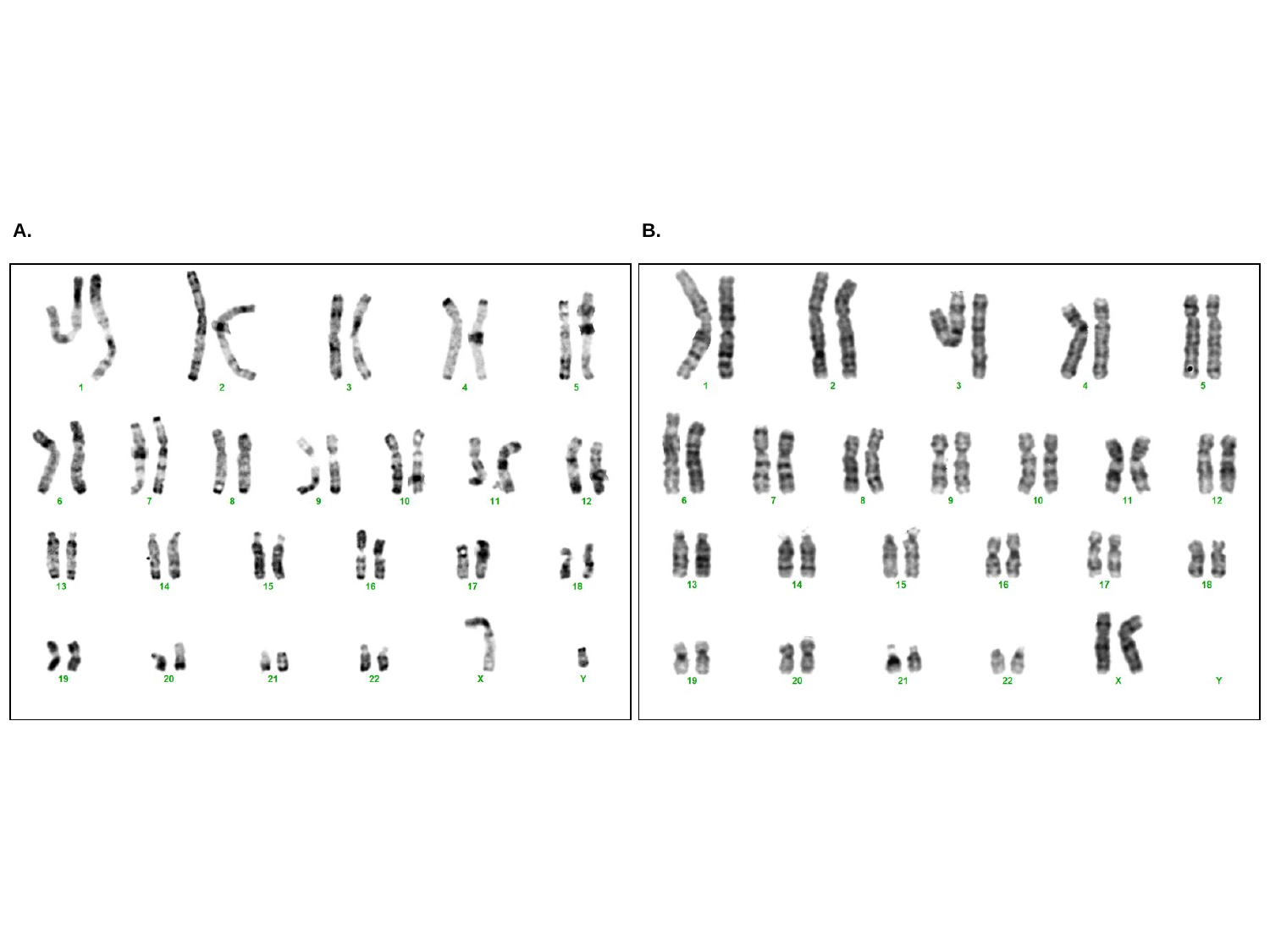

A.
B.

Supplement: Additional file 3: Figure S2. — Conventional cytogenetic analysis. A. G-banded karyotype of H1 hESC line at passage 64 showing a normal male karyotype 46,XY. B. G-banded karyotype of H9 hESC line at passage 59 showing a normal female karyotype 46,XX. [file 13039_2015_133_MOESM3_ESM.ppt]

## Slide 1
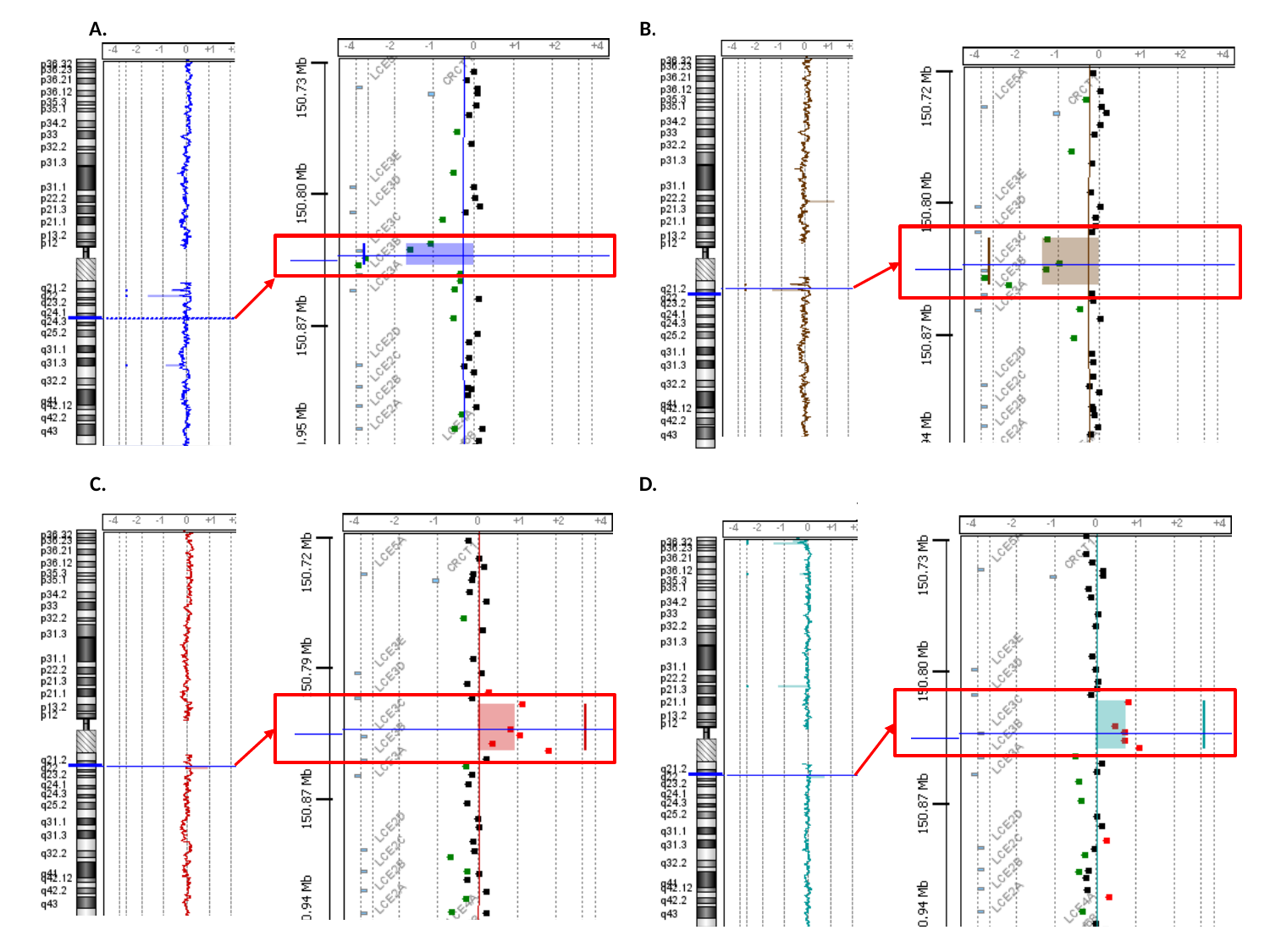

A.
B.
!!
C.
D.

Supplement: Additional file 4: Figure S3. — Chromosome 1 profile from array-CGH analysis showing recurrent variation on 1q21.3 region. Profiles are illustrated for H1p56 (a; early and manual passage), H1p159 (b; late and enzymatic passage), H9p30 (c; early and manual passage) and H9p87 (d; late and enzymatic passage). For each sample, whole chromosome 1 profile showing the interstitial variation is presented on the left panel; and the 1q21.3 deletion (a and b) or duplication (c and d) is zoomed in the right panel. [file 13039_2015_133_MOESM4_ESM.pptx]
