# Supplementary material for: Genomic instability of human embryonic stem cell lines using different passaging culture methods
Source: Mol Cytogenet. 2015 Apr 23;8:30. doi: 10.1186/s13039-015-0133-8 (PMC4456787; doi:10.1186/s13039-015-0133-8)
Supplement: Additional file 2: Table S5. — Passage number at which analysis were done in hESC lines H1 and H9. List of the passage number using either manual or enzymatic (collagenase IV) methods at with flow cytometry, karyotype, array-CGH and teratoma analysis were realized of the hESC lines H1 and H9. [file 13039_2015_133_MOESM2_ESM.docx]

Additional Table 5. Passage number at which analysis were done in hESC lines H1 and H9.

| **hESC line** | **Passage number** | **Method** | **Flow cytometry** | **Karyotype and FISH** | **Array-CGH** | **Teratoma assay** |
| --- | --- | --- | --- | --- | --- | --- |
| **H1** | p30 | collagenase | x | x |  |  |
|  | p32 | manual | x | x |  |  |
|  | p44 | manual | x | x |  | x |
|  | p51 | manual | x | x |  |  |
|  | p56 | collagenase, manual | x | x | x |  |
|  | p61 | collagenase, manual | x | x |  |  |
|  | p63 | collagenase | x | x |  | x |
|  | p64 | collagenase, manual | x | x |  |  |
|  | p139 | collagenase | x | x |  |  |
|  | p159 | collagenase | x | x | x |  |
| **H9** | p28 | manual | x | x |  |  |
|  | p29 | manual | x | x |  |  |
|  | p30 | collagenase, manual | x | x | x |  |
|  | p35 | collagenase, manual | x | x |  |  |
|  | p38 | manual | x | x |  | x |
|  | p40 | manual | x | x |  |  |
|  | p40 | collagenase | x | x |  | x |
|  | p51 | manual | x | x |  |  |
|  | p55 | manual | x | x |  |  |
|  | p59 | manual | x | x |  |  |
|  | p60 | collagenase, manual | x | x |  |  |
|  | p69 | collagenase, manual | x | x |  |  |
|  | p77 | collagenase | x | x |  |  |
|  | p87 | collagenase | x | x | x |  |
