# Supplementary material for: Genomic instability of human embryonic stem cell lines using different passaging culture methods
Source: Mol Cytogenet. 2015 Apr 23;8:30. doi: 10.1186/s13039-015-0133-8 (PMC4456787; doi:10.1186/s13039-015-0133-8)
Supplement: Additional file 5: Table S1. — Genes related to development. List of genes related to development located in genomic variations corresponding to Figure 3B. Chromosomal position, gene symbol and encoded protein are noted. G, gain; L, loss. [file 13039_2015_133_MOESM5_ESM.docx]

Additional Table 1. Genes related to development.

| **Chromosomal region** | **Gene** | **Encoded protein** | **H1 M early** | **H1 E early** | **H1 E late** | **H9 M early** | **H9 E early** | **H9 E late** |
| --- | --- | --- | --- | --- | --- | --- | --- | --- |
| **1p36.23** | RERE | arginine-glutamic acid dipeptide repeats | - | - | - | - | G | - |
| **1q25.2** | LHX4 | LIM homeobox 4 | - | - | - | - | G | - |
| **9q34.3** | NOTCH1 | Notch 1 | - | - | - | - | - | - |
| **14q32.33** | JAG2 | jagged 2 | - | - | - | - | - | - |
| **16q24.3** | SNAI3 | snail homolog 3 | - | - | - | - | - | - |
| **17q12** | MMP28 | matrix metallopeptidase 28 | - | - | - | - | - | L |
| **18q11.2** | CHST9 | carbohydrate sulfotransferase 9 | L | - | - | - | - | - |
| **20p11.21** | PLAGL2 | pleiomorphic adenoma gene-like 2 | - | - | G | - | - | - |
| **22q13.31** | CELSR1 | cadherin, EGF LAG seven-pass G-type receptor 1 | - | - | - | - | - | L |
