# Supplementary material for: Genomic instability of human embryonic stem cell lines using different passaging culture methods
Source: Mol Cytogenet. 2015 Apr 23;8:30. doi: 10.1186/s13039-015-0133-8 (PMC4456787; doi:10.1186/s13039-015-0133-8)
Supplement: Additional file 6: Table S2. — Genes related to cell cycle, growth and apoptosis. List of genes related to cell cycle, growth or apoptosis located in genomic variations corresponding to Figure 3B. Chromosomal position, gene symbol and encoded protein are noted. G, gain; L, loss. [file 13039_2015_133_MOESM6_ESM.docx]

Additional Table 2. Genes related to cell cycle, growth and apoptosis.

| **Chromosomal region** | **Gene** | **Encoded protein** | **H1 M early** | **H1 E early** | **H1 E late** | **H9 M early** | **H9 E early** | **H9 E late** |
| --- | --- | --- | --- | --- | --- | --- | --- | --- |
| **1p36.23** | RERE | arginine-glutamic acid dipeptide repeats | - | - | - | - | G | - |
| **1q25.2** | QSOX1 | quiescin Q6 sulfhydryl oxidase 1 | - | - | - | - | G | - |
| **2q37.3** | GPC1 | glypican 1 | - | - | - | - | - | L |
| **4p16.3** | FGFR3 | fibroblast growth factor receptor 3 | - | - | - | - | - | - |
|  | FGFRL1 | fibroblast growth factor receptor-like 1 | - | - | - | - | - | - |
|  | SLBP | stem-loop binding protein | - | - | - | - | - | - |
|  | CTBP1 | c-terminal binding protein 1 | - | - | - | - | - | - |
| **7p22.1** | RADIL | ras association and DIL domains | - | - | - | - | - | - |
| **7q36.3** | PTPRN2 | protein tyrosine phosphatase, receptor type, N polypeptide 2 | - | - | - | - | - | - |
| **8q24.22** | NDRG1 | N-myc downstream regulated 1 | - | - | - | - | - | - |
| **8q24.3** | PTP4A3 | protein tyrosine phosphatase type IVA, member 3 | - | - | - | - | - | - |
| **9q22.2** | SYK | spleen tyrosine kinase | L | - | - | - | - | - |
| **9q34.2q34.3** | ANAPC2 | anaphase promoting complex subunit 2 | - | - | - | - | - | - |
|  | WDR5 | WD repeat domain 5 | - | - | - | - | - | - |
|  | CARD9 | caspase recruitment domain family, member 9 | - | - | - | - | - | - |
|  | TRAF2 | TNF receptor-associated factor 2 | - | - | - | - | - | - |
| **11p15.5** | DUSP8 | dual specificity phosphatase 8 | - | - | - | - | - | - |
|  | INS | insulin | - | - | - | - | - | - |
|  | IGF2 | insulin-like growth factor 2 | - | - | - | - | - | - |
|  | CD81 | CD81 | - | - | - | - | - | - |
|  | CD151 | CD151 | - | - | - | - | - | - |
|  | TSPAN4 | tetraspanin 4 | - | - | - | - | - | - |
|  | NLRP6 | NLR family, pyrin domain containing 6 | - | - | - | - | - | - |
|  | LRDD | p53-induced death domain protein | - | - | - | - | - | - |
| **14q22.1** | CDKL1 | cyclin-dependent kinase-like 1 | - | - | - | - | - | - |
| **14q23.2** | RHOJ | ras homolog gene family, member J | - | - | - | G | - | G |
| **14q32.33** | CDCA4 | cell division cycle associated 4 | - | - | - | - | - | - |
|  | GPR132 | G protein-coupled receptor 132 | - | - | - | - | - | - |
|  | SIVA1 | SIVA1, apoptosis-inducing factor | - | - | - | - | - | - |
| **17p11.2** | RASD1 | RAS, dexamethasone-induced 1 | - | - | - | - | - | L |
| **20p11.21** | BCL2L1 | BCL2-like 1 | - | - | G | - | - | - |
|  | PDRG1 | p53 and DNA-damage regulated 1 | - | - | G | - | - | - |
| **Xq28** | FAM58A | family with sequence similarity 58, member A | - | - | - | - | - | - |
