# Supplementary material for: Genomic instability of human embryonic stem cell lines using different passaging culture methods
Source: Mol Cytogenet. 2015 Apr 23;8:30. doi: 10.1186/s13039-015-0133-8 (PMC4456787; doi:10.1186/s13039-015-0133-8)
Supplement: Additional file 7: Table S3. — Genes related to tumorigenesis. List of genes related to tumorigenesis located in genomic variations corresponding to Figures 3B and 3C. Chromosomal position, gene symbol and encoded protein are noted. G, gain; L, loss. [file 13039_2015_133_MOESM7_ESM.docx]

Additional Table 3. Genes related to tumorigenesis.

| **Chromosomal region** | **Gene** | **Encoded protein** | **H1 M early** | **H1 E early** | **H1 E late** | **H9 M early** | **H9 E early** | **H9 E late** | **Role** |
| --- | --- | --- | --- | --- | --- | --- | --- | --- | --- |
| **3q21.3** | PLXNA1 | plexin A1 | - | - | - | - | - | - | **ONCOGENES** |
| **4p16.3** | TACC3 | transforming, acidic coiled-coil containing protein 3 | - | - | - | - | - | - |  |
| **8q24.3** | PSCA | prostate stem cell antigen | - | - | - | - | - | - |  |
| **9q34.3** | EGFL7 | EGF-like-domain multiple 7 | - | - | - | - | - | - |  |
|  | VAV2 | vav 2 guanine nucleotide exchange factor | - | - | - | - | - | - |  |
| **11p15.5** | HRAS | v-Ha-ras Harvey rat sarcoma viral oncogene homolog | - | - | - | - | - | - |  |
| **11p15.5** | IFITM1-3,5 | interferon induced transmembrane protein 1-3,5 | - | - | - | - | - | - |  |
| **11q13.4** | RAB6A | member RAS oncogene family | G | G | - | - | - | - |  |
| **14q23.2** | CTSD | cathepsin D | - | - | - | - | - | - |  |
|  | RHOJ | ras homolog gene family, member J | - | - | - | G | - | G |  |
| **14q23.3** | FUT8 | fucosyltransferase 8 (alpha (1,6) fucosyltransferase) | - | - | - | G | - | G |  |
| **16q22.1** | ST3GAL2 | ST3 beta-galactoside alpha-2,3-sialyltransferase 2 | - | - | - | - | G | - |  |
| **17q12** | MMP28 | matrix metallopeptidase 28 | - | - | - | - | - | L |  |
| **22q11.22** | SH3GL1 | SH3-domain GRB2-like 1 | - | - | - | - | L | - |  |
|  | CHAF1A | chromatin assembly factor 1, subunit A (p150) | - | - | - | - | L | - |  |
| **Xp22.2** | CTPS2 | CTP synthase II | - | - | - | - | G | - |  |
| **7p22.3** | MAD1L1 | MAD1 mitotic arrest deficient-like 1 | - | - | - | - | L | L | **TUMOR SUPPRESSOR GENES** |
| **7q31.2** | CAV1 | Caveolin 1 | L | - | - | - | - | - |  |
| **7q36.3** | PTPRN2 | protein tyrosine phosphatase, receptor type, N polypeptide 2 | - | - | - | - | - | - |  |
| **8p12** | DUSP26 | dual specificity phosphatase 26 | - | - | - | - | G | - |  |
| **8q24.12** | DEPDC6 | DEP domain containing MTOR-interacting protein | - | - | - | - | G | - |  |
| **8q24.3** | SLURP1 | secreted LY6/PLAUR domain containing 1 | - | - | - | - | - | - |  |
|  | BAI1 | brain-specific angiogenesis inhibitor 1 | - | - | - | - | - | L |  |
| **9q22.2** | SYK | Spleen tyrosine kinase | L | - | - | - | - | - |  |
| **9q34.3** | QSOX2 | quiescin Q6 sulfhydryl oxidase 2 | - | - | - | - | - | - |  |
| **14q23.2** | TNNI2 | troponin I type 2 | - | - | - | - | - | - |  |
|  | TSSC4 | tumor suppressing subtransferable candidate 4 | - | - | - | - | - | - |  |
| **16q24.3** | CBFA2T3 | core-binding factor, runt domain, alpha subunit 2; translocated to, 3 | - | - | - | - | - | - |  |
| **17p13.3** | RPH3AL | rabphilin 3A-like | - | - | - | - | G | - |  |
| **22q11.22** | RTDR1 | rhabdoid tumor deletion region gene 1 | - | - | - | - | - | - |  |
| **Xp11.23** | UXT | ubiquitously-expressed transcript | - | - | - | - | - | - |  |
| **1p36.32** | PRDM16 | PR domain containing 16 | - | - | - | - | - | L | **OTHERS** |
| **17p13.3** | FUK | fucokinase | - | - | - | - | G | - |  |
| **19p13.3** | MLLT1 | lymphoid or mixed-lineage leukemia translocated to, 1 | - | - | - | - | - | - |  |
