# Supplementary material for: Genomic instability of human embryonic stem cell lines using different passaging culture methods
Source: Mol Cytogenet. 2015 Apr 23;8:30. doi: 10.1186/s13039-015-0133-8 (PMC4456787; doi:10.1186/s13039-015-0133-8)
Supplement: Additional file 8: Table S4. — Genes related to syndrome and disease. List of genes related to syndrome or human disease located in genomic variations corresponding to Figure 3B. Chromosomal position, gene symbol and encoded protein are noted. G, gain; L, loss. [file 13039_2015_133_MOESM8_ESM.docx]

Additional Table 4. Genes related to syndrome and disease.

| **Chromosomal region** | **Gene** | **Encoded protein** | **H1 M early** | **H1 E early** | **H1 E late** | **H9 M early** | **H9 E early** | **H9 E late** |
| --- | --- | --- | --- | --- | --- | --- | --- | --- |
| **1p36.12** | C1QA-C | complement component 1, q subcomponent | -  -  - | -  -  - | -  -  - | -  -  - | -  -  - | L |
| **2p21** | LRPPRC | leucine-rich PPR-motif containing | -  -  - | L  -  - | -  -  - | -  -  - | -  -  - | -  -  - |
| **4p16.3** | PDE6B | phosphodiesterase 6B cGMP-specific rod beta | -  -  - | -  -  - | -  -  - | -  -  - | -  -  - | -  -  - |
| **4q13.3** | SNCA | synuclein, alpha | -  -  - | -  -  - | -  -  - | -  -  - | -  -  - | -  -  - |
| **6p24.3** | TFAP2A | transcription factor AP-2 alpha | -  -  - | -  -  - | -  -  - | -  -  - | G | -  -  - |
| **6q26** | PACRG | PARK2 co-regulated | -  -  - | -  -  - | -  -  - | -  -  - | -  -  - | -  -  - |
| **8q24.3** | PUF60 | poly-U binding splicing factor 60KDa | -  -  - | -  -  - | -  -  - | -  -  - | -  -  - | L |
| **17p11.2** | PEMT | phosphatidylethanolamine N-methyltransferase | -  -  - | -  -  - | -  -  - | -  -  - | -  -  - | L |
